# The management of paediatric diaphyseal femoral fractures: a modern approach

**DOI:** 10.1007/s11751-016-0258-2

**Published:** 2016-07-11

**Authors:** Al-achraf Khoriati, Carl Jones, Yael Gelfer, Alex Trompeter

**Affiliations:** 1 St George’s Hospital, 68 Daybrook Road, London, SW19 3DH UK; 2 Royal County Surrey, Guildford, UK

**Keywords:** Paediatric, Femur, Fracture, Management, Review, Trauma, Evidence

## Abstract

The definitive treatment of paediatric femoral diaphyseal fractures remains controversial. Modalities of treatment vary mostly according to age, with fracture pattern and site having a lesser impact. Current evidence is reflective of this variation with most evidence cited by the American Academy of Orthopedic Surgeons being level 4 or 5. The authors present a review of the most up-to-date evidence relating to the treatment of these fractures in each age group. In an attempt to clarify the current trends, we have produced an algorithm for decision-making based on the experience from our own tertiary referral level 1 major trauma centre.

## Introduction

Femoral fractures are among the most common fractures of long bones [1]. The management of paediatric femoral fractures depends primarily on the age of the child although the bone age and size of a child may determine the choice of treatment [2]. The choice of management may also be determined by surgical experience and local trends in practice. Non-operative management plays a role in some cases still though current practice has veered towards operative fixation as it allows early mobilisation and shorter hospital stays.

In this review, the authors provide a narrative review of management techniques for paediatric diaphyseal femoral fractures. The benefits and limitations of each technique will be considered as well as the published evidence. An algorithm is provided for decision-making based on the experience gathered from our own tertiary referral level 1 major trauma centre which provides a pathway for the management of these fractures.

### Epidemiology

Epidemiological studies on paediatric fractures of the femur are rare in the UK. The largest of these is a study of 3272 children under the age of 16 [1]. Between 1991 and 2002, the incidence of these fractures decreased from 0.33 to 0.22 femoral fractures/1000/year. It is speculated that this may be due to improved road safety or reduced levels of physical activity and outdoor play time in recent years [1].

While the incidence is equal in both genders in the first year of life, it always was found to increase in boys thereafter; boys are 4.7 times more likely to have sustained a femoral fracture by the age of 14 [1]. The difference in risk with gender has been the subject of much debate, and there is, as yet, no evidence to support any particular explanation.

### Non-accidental injury (NAI)

The single best predictor of whether or not a paediatric femoral fracture is caused non-accidentally is the child’s ability to walk [3]. Although fracture patterns may vary, no individual fracture type can distinguish an accidental from a non-accidental injury. History taking is key and the plausibility of the story presented by a child’s parents must be thoroughly assessed [4]. Further investigations into other causes of the injury (i.e., metabolic, mechanical or medical) must be carried out as these may help exclude a non-accidental cause.

### Anatomy

In contrast to adults, the immature skeleton is characterised by the presence of open physes, thicker periosteum, and a different biomechanical behaviour in response to loading. As proximal and distal growth plates are both placed at risk during the insertion of intramedullary fixation, they must be protected to prevent varying degrees of growth disturbance.

The paediatric femur, in contrast to the adult femur, has a high capacity for remodelling and as such will tolerate up to 25 degrees of angulation in any plane [5]. Rotational deformity is less well tolerated although studies have reported that up to 25 % of malrotation is accepted [6]. A shortening of up to 1 cm in those under the age of 10 is accepted due to overgrowth which is caused by the vessel-rich periosteum being stimulated in response to local injury [2].

### Aetiology

The aetiology of femoral diaphyseal fractures varies with the age of the patient. Femoral fractures in adolescents and older children are more likely to be caused by a high-energy injury, while, in younger children, falls from standing height or from playground equipment are more likely [7].

Twelve per cent of femoral fractures in children aged 4 or less are pathological [8]. Common causes include nonossifying fibroma, fibrous dysplasia, aneurysmal and unicameral bone cyst and osteosarcoma [9]. Stress fractures of the femoral diaphysis are rare in children and account for only 4 % of all paediatric stress fractures [2].

### Classification

**Fig. 1:**
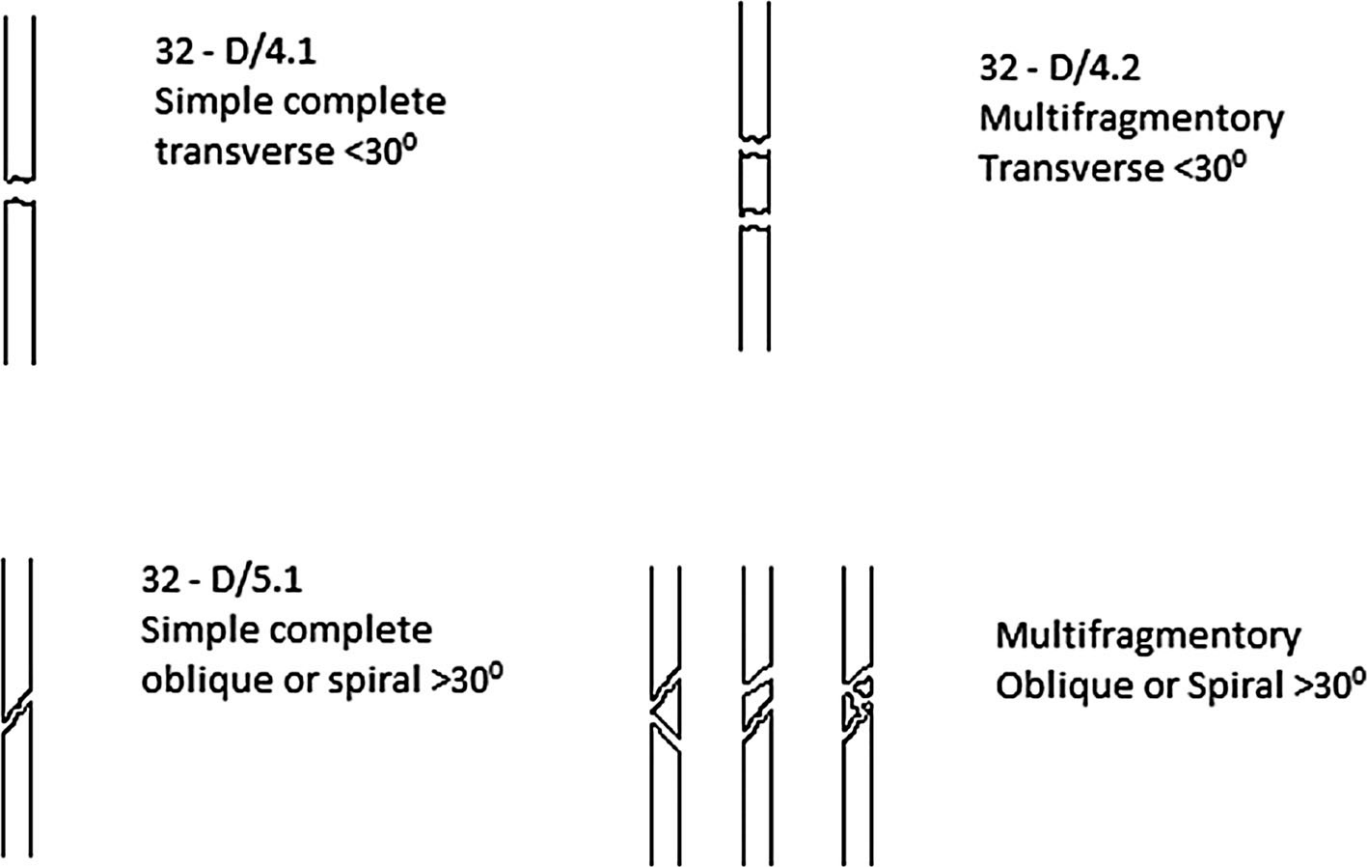
Paediatric diaphyseal classification system

This is based on the Müller AO classification for adults and considers features which are child specific (Fig. 1). In contrast to adult fractures, grading A, B and C has been replaced with D, M and E denoting diaphysis, metaphysis and epiphysis, respectively. Severity grading has been added to differentiate simple (.1) and a wedge, complex or multifocal entry (.2) fracture.

### Principles of fracture treatment and factors influencing treatment

The aim of fracture treatment in children is the restoration of function and a normal level of activity as quickly as possible with the minimum physical and psychological distress. Dameron et al. [10] outlined 6 key principles for the treatment of paediatric diaphyseal fractures:

1. The simplest treatment is the best treatment.
2. The initial treatment should be definitive whenever possible.
3. Anatomic reduction was not required for perfect function.
4. Alignment must be restored, especially rotational alignment.
5. The more growth that remained, the more remodelling was available.
6. The limb should be immobilised in a splint until definitive treatment had been instituted.

While these principles still hold true today, a number of other factors must be considered:

1. The age and weight of a child.
2. The fracture configuration.
3. The experience of the treating surgeon.
4. The availability/cost of treatment.

A child’s potential for remodelling varies with age. The potential for correction of deformity is great in infancy but largely disappears by the beginning of adolescence [11]. The biomechanical behaviour of a heavy teenager’s bone is often far closer to that of an adult patient than a child [12].

Social circumstances influence these principles. The modern family requires two working partners; it is difficult for a parent to take time off work for an extended period. Furthermore, educational needs of children have changed with modern curriculums unable to cater for prolonged periods of absence from study. Finally, healthcare resources are stretched with many facilities unable to provide the staff or facilities allowing for prolonged hospital admission.

### Treatment of fractures by age group

#### The neonate and infant

Femoral fractures that occur during birth are rare [13]. Neonates can be managed with immobilisation in a Pavlik harness for up to 3 weeks. Callus formation occurs quickly, and there are few long-term consequences observed [14].

A femoral fracture in an infant is highly suspicious of NAI given that they are non-ambulatory and must be investigated thoroughly. Management options for the fracture in this age group tend to be non-invasive and include either traction or hip spica casting. Often a combination of both is preferred as spica application may require anaesthesia often and a paediatric anaesthetist may not always be available immediately. Callus forms rapidly in the infant, and femoral shaft fractures may become relatively stable after the first week in traction. Spica application may occur after this stage without the need for an anaesthetic.

Skin traction in smaller children (<12 kg) should be in the form of gallows traction. The use of this technique in larger children is not recommended as it has been associated with compartment syndrome, Volkmann’s contracture and common peroneal nerve palsy [14]. In heavier infants, greater patient comfort and better control of the fracture can be achieved by using Hamilton-Russell skin traction. This method of traction with leg support can be also used to control femoral rotation.

A considerable amount of shortening and angulation is tolerated in this age group (15 mm of shortening and 30 degrees of angulation) [15]. Rotational deformity is less common and is not well tolerated.

#### Young children and toddlers aged 18 months to 5 years

Femoral fractures in this age group are most likely caused by a simple fall from standing height. A systematic review [16] indicated that the NAI accounted for 0.5 % of these injuries in this age group compared to 11 % in infants. Non-invasive management is still preferred in this age group.

**Fig. 2:**
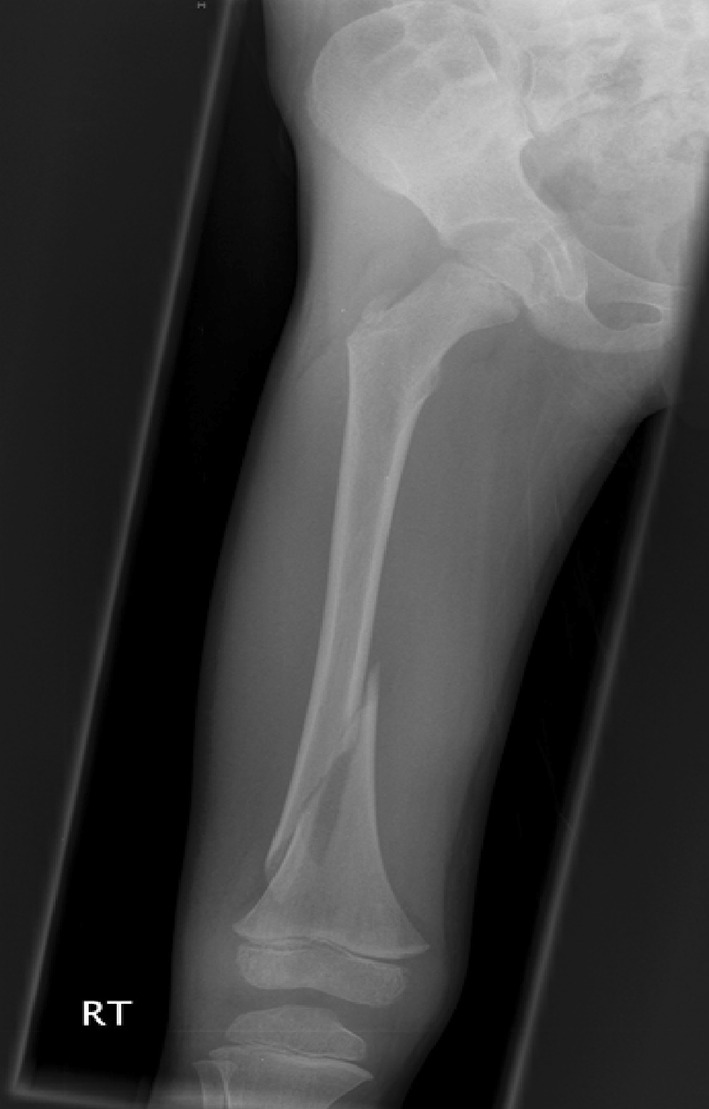
AP radiograph demonstrating a distal 1/3 spiral femoral diaphyseal fracture in a 6-year-old child

**Fig. 3:**
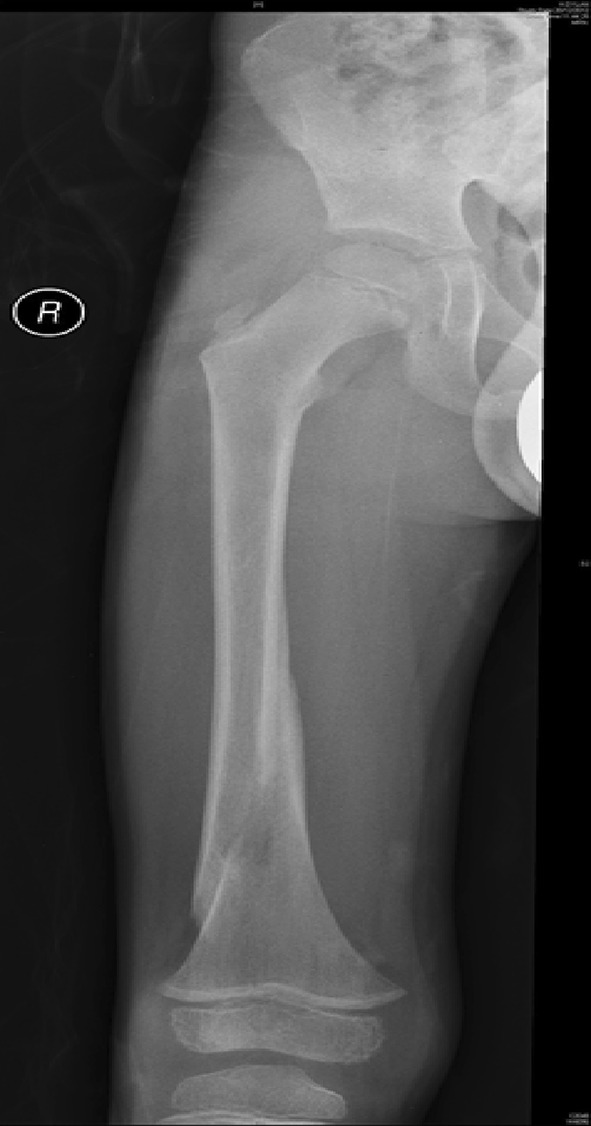
AP radiograph of the same child as shown in Fig. 2, taken at 8 weeks, showing solid union and acceptable alignment after an initial treatment of 3- to 4-week in-line traction

**Fig. 4:**
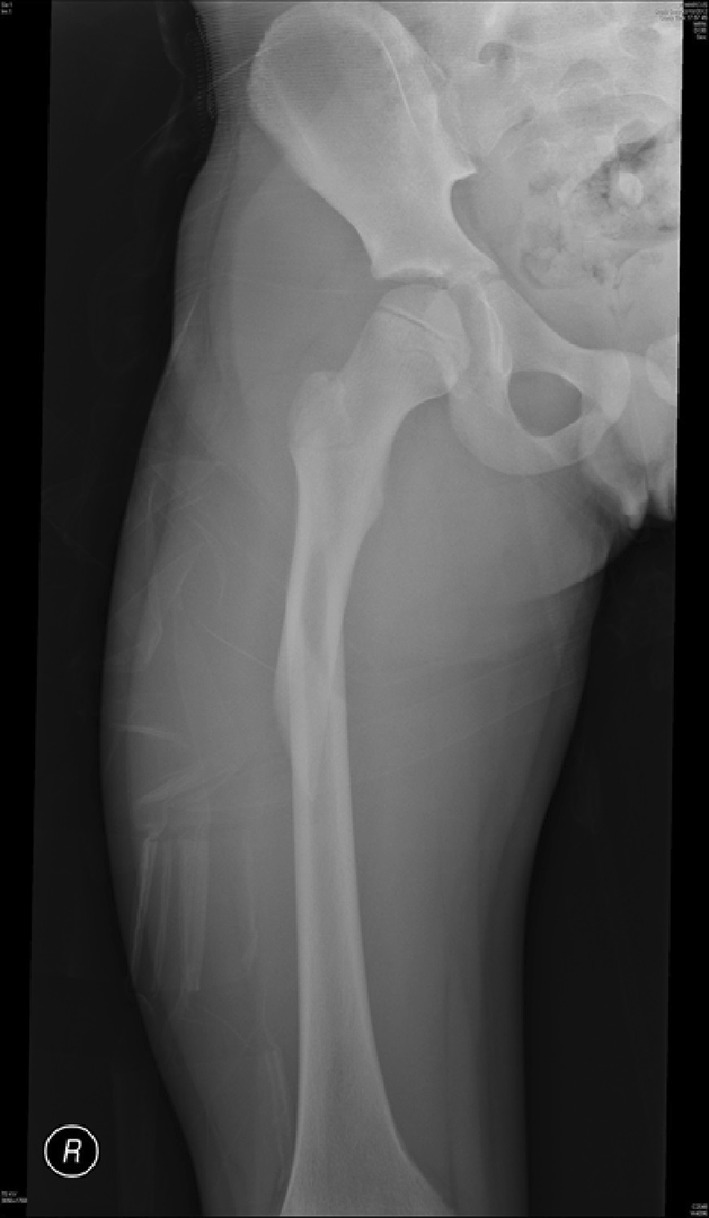
AP radiographs demonstrating a proximal 1/3 spiral femoral diaphyseal fracture in a 6-year-old child

**Fig. 5:**
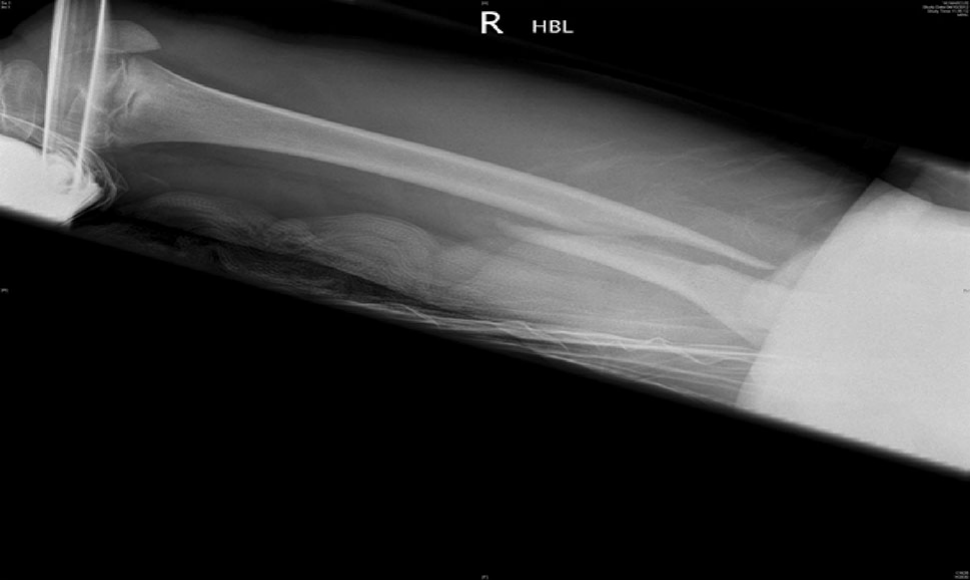
Lateral radiographs demonstrating a proximal 1/3 spiral femoral diaphyseal fracture in a 6-year-old child

**Fig. 6:**
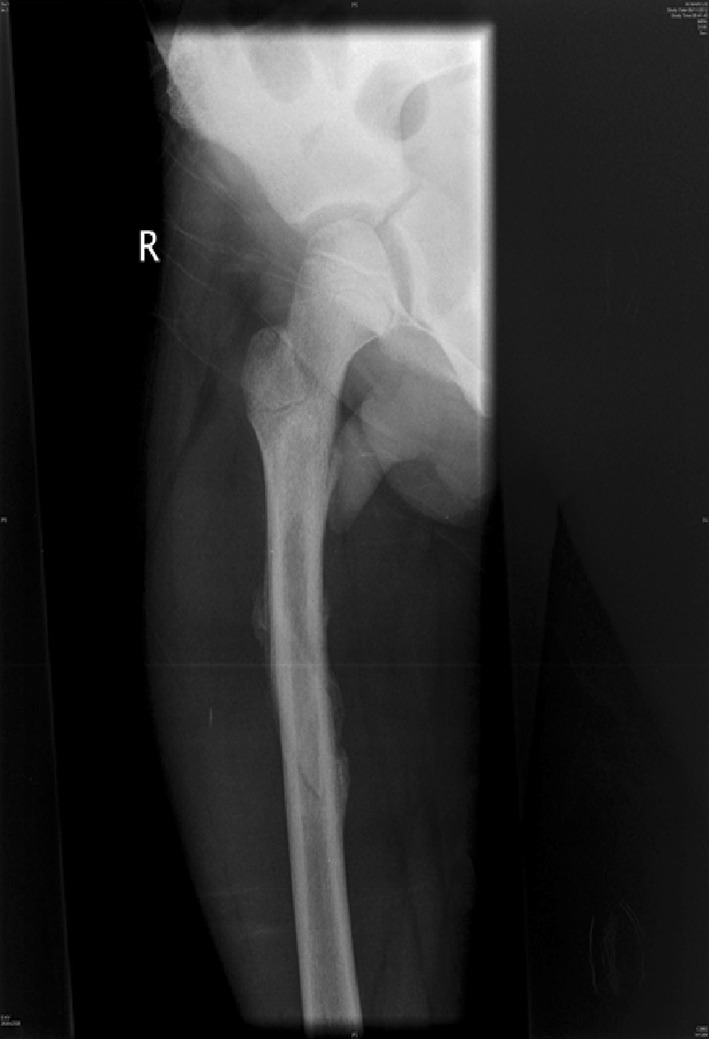
After 4 weeks in traction, a healthy callus is seen to form in children aged 5–12 years

**Fig. 7:**
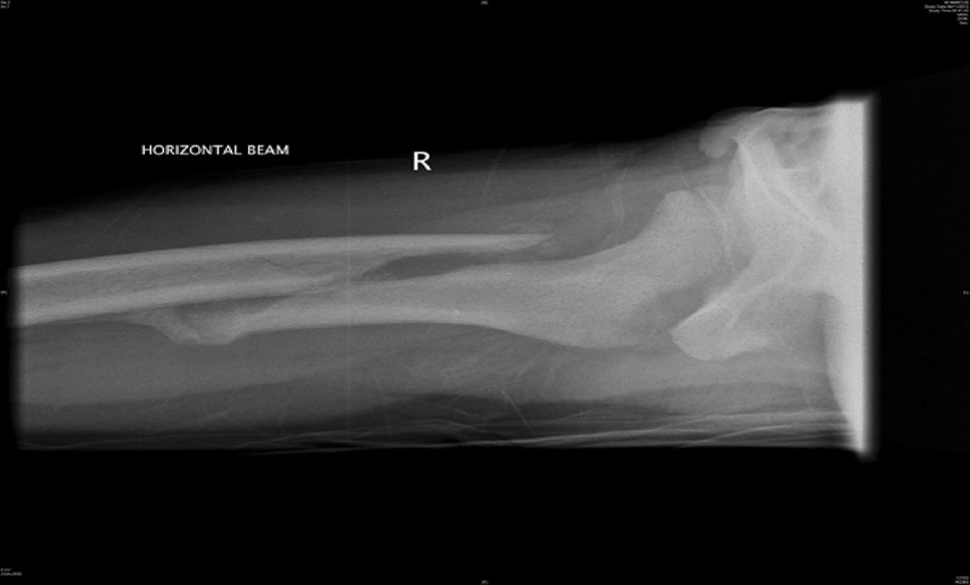
After 4 weeks in traction, a healthy callus is seen to form in children aged 5–12 years

Traction is the preferred method in most instances (see Figs. 2, 3, 4, 5, 6, 7). The use of fixed traction systems such as Thomas or Liston splints may cause pressure injury to the skin [17] and should be used as temporary measures only. Balanced traction systems are suitable for definitive management. Hamilton-Russell skin traction is the method of choice [7, 14, 18]. One pound of weight and 1 week of traction are usually required per year of age [14]. However, it is relatively complex and most centres will no longer have the expertise to apply it. Straight line or in-line traction is easier to apply generally and more common.

**Fig. 8:**
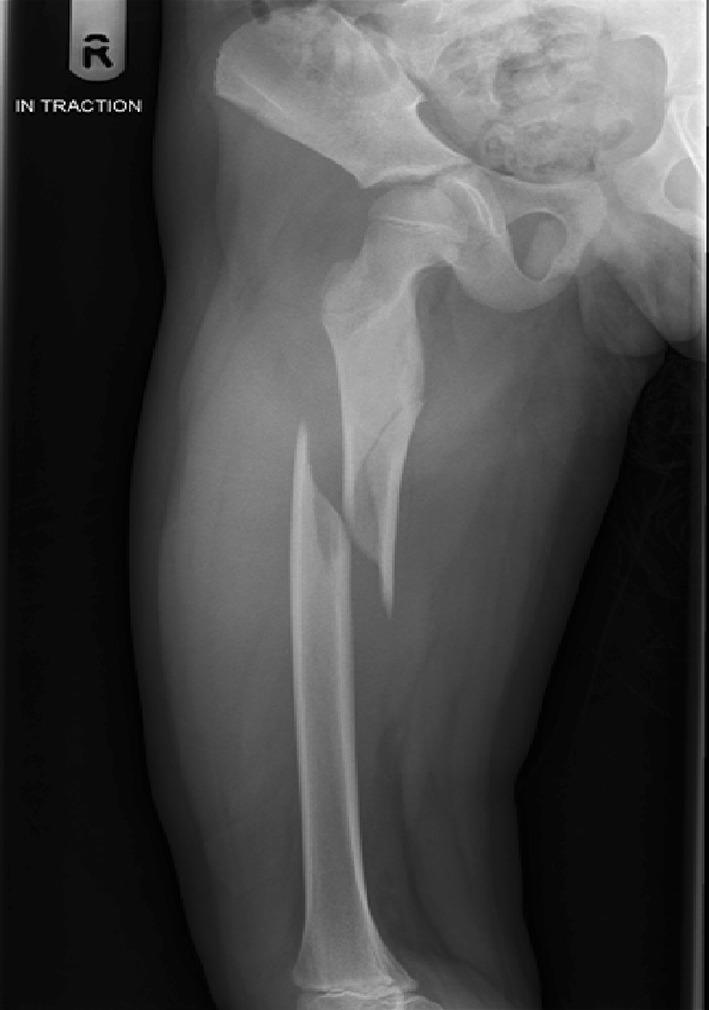
AP radiograph demonstrating a proximal third diaphyseal fracture of the femur in an 8-year-old. Note the spiral fracture with a butterfly fragment—elastic nails may be unstable here

Hip spica casting may be initiated following an initial period of traction. This reduces the risk of malunion—a recognised complication associated with spica casting [19, 20]. In this age group, the femur still retains a good capacity for remodelling. Fifteen degrees of varus or valgus angulation and 25 degrees of flexion or extension may be tolerated [5]. Compartment syndrome is a recognised complication of spica management, and care must be taken in order to avoid overzealous moulding of casts [15]. Spica casting may be contraindicated in instances when the skin (dermatological conditions) or the soft tissues (open fractures) may be compromised.

As an increasing period of immobilisation is required for non-operative management in older children, management options begin to veer towards surgery as it allows for earlier return of function and reduces impact on modern family life. Traction, splinting and spica casting all remain options although some authors argue that the latter is inappropriate in patients over 4 years [14]. Skin traction carries the risk of pressure sores, whereas skeletal traction may carry the risk of damaging the proximal tibia [7] or distal femoral physes [21]. If traction is used in the acute setting, there is little evidence to support skin traction over skeletal traction and vice versa [22]. Limb shortening remains an issue with spica casting [23]. Some shortening may be desirable to accommodate for overgrowth, but children in this age group managed with spica casting should undergo regular clinical and radiological review in order to detect unresolved length discrepancies which can be unacceptable, particularly in older children.

### Plate fixation

**Fig. 9:**
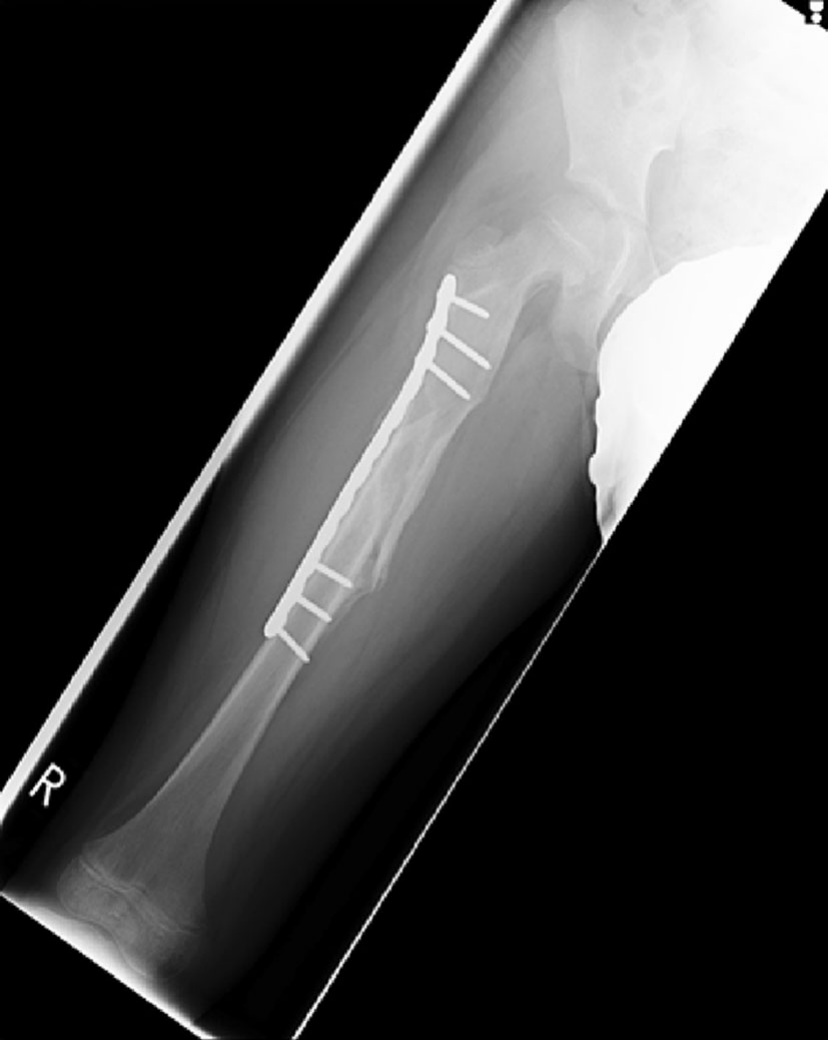
AP radiograph showing the patient from Fig. 8, treated with a submuscular 3.5-mm bridge plate. At just 8 weeks, there is abundant callus and the patient is fully weight bearing

The publication of long-term follow-up outcome studies and reports of complications with the use of other treatment modalities has led to a resurgence of interest in femoral plating [24] which was reserved traditionally for use in polytrauma patients, in adolescents, or for stabilising fractures too proximal to manage with intramedullary nails [14] (Fig. 9).

The use of plates to treat fractures in such young patients is favoured due to the fact that these fractures heal rapidly and the complication of plate failure, which is seen in adults, is rarely observed [25]. The use of compression plating is reported to lead to fracture union within 8–11 weeks [25, 26]. Complications associated with traditional plating methods include the extensive amount of exposure needed to achieve anatomic reduction and the subsequent soft tissue damage and periosteal stripping. High infection rates were reported in the earlier literature. The removal of plates remains an issue as screw holes left in the femur create stress risers within it [2].

There has been a recent trend in both paediatric and adult trauma towards the management of fractures of the femoral diaphysis with minimally invasive bridge plates. This method carries the advantage of less soft tissue damage and a smaller scar. It has been suggested that bridge plating is superior to conventional plating because it preserves the periosteal blood supply and disturbs the soft tissue envelope minimally [27]. Kanlic et al. [28] proposed the concept that submuscular bridge plating combined the advantages of both conservative and surgical treatment methods. With bridge plating, the preservation of biology at the fracture site was achieved without sacrificing alignment, early mobilisation and ease of care. Minimally invasive or submuscular techniques have a role to play in the management of comminuted fractures although they can be used in most fracture patterns. Small plate (3.5 mm) systems are used typically in children as opposed to the larger 4.5 mm systems employed in adults. Restoration of leg length remains an issue highlighted in the literature with the majority of leg length inequality thought to be created at the time of the operation [29].

#### Intramedullary fixation

**Fig. 10:**
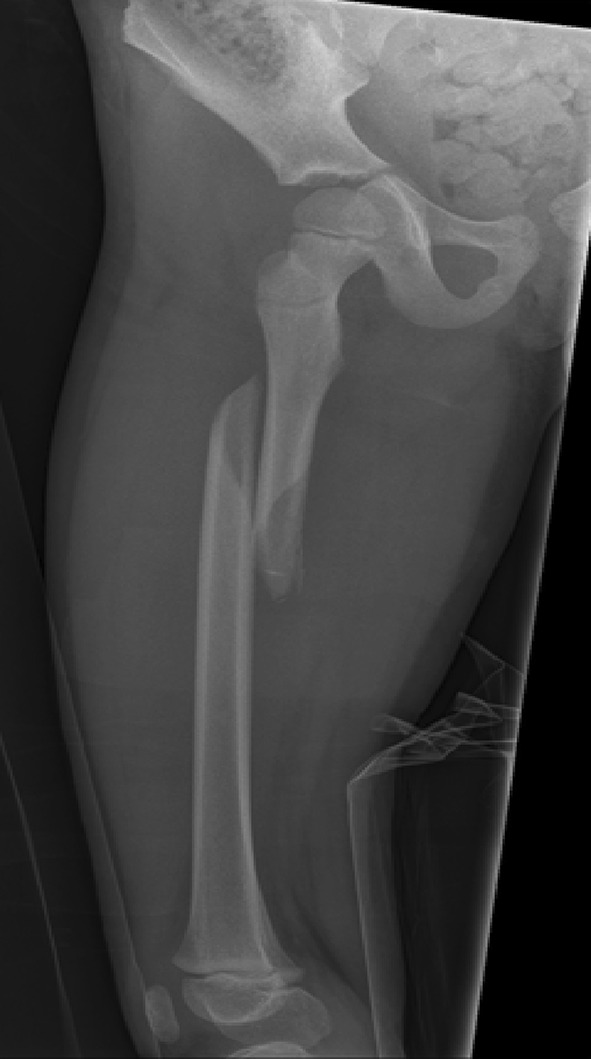
AP radiograph demonstrating a spiral femoral fracture ideal for treatment with flexible nails

**Fig. 11:**
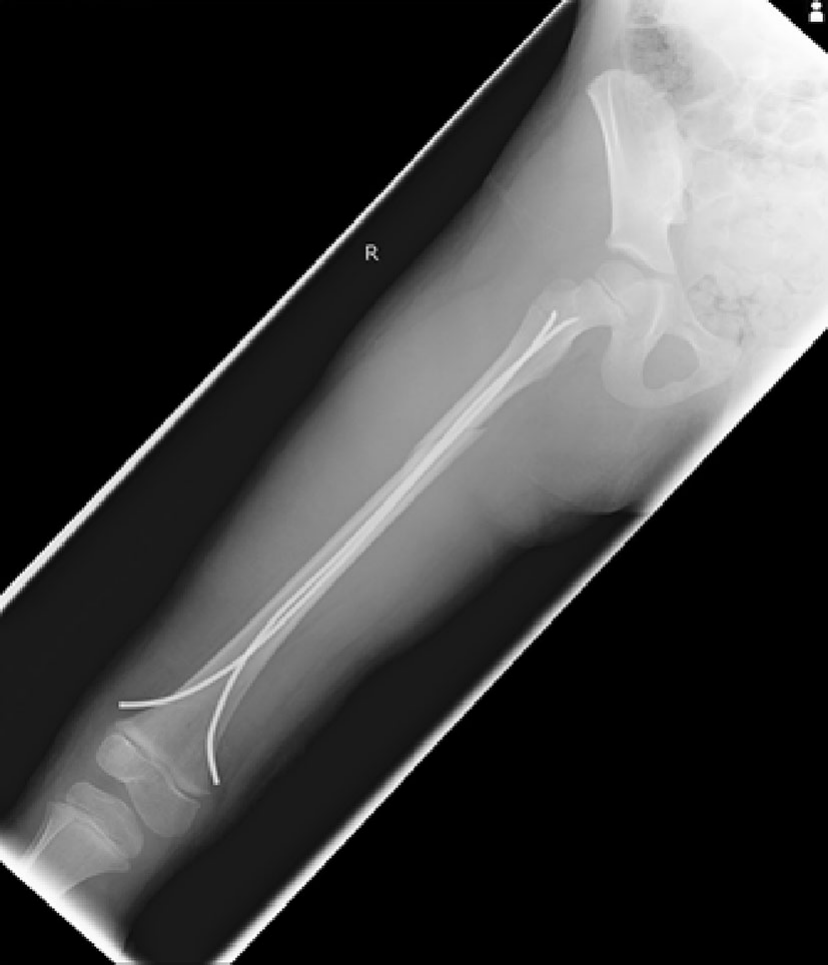
AP radiograph showing satisfactory restoration of length, rotation and alignment with the use of titanium elastic nails, of the fracture in Fig. 10

Flexible intramedullary nailing using either stainless steel or titanium nails has increased in popularity and is now the technique of choice in the management of most femoral diaphyseal fractures (Figs. 10, 11) as it is minimally invasive, offers a shorter hospital stay and allows earlier mobilisation. Weight bearing is restricted initially and advanced to partial from 2 to 3 weeks. Some advocate a more cautious approach in patients with unstable fracture patters [7].

The two types of nail differ slightly in their method of use. Titanium nails are more elastic, and the balanced forces of each nail are used to stabilise the fracture. Stainless steel nails, such as the Ender nail, are more rigid and are used to fill the canal. Due to their elasticity, titanium nails are thought to promote callus formation by limiting stress shielding [30–32] and allow for enough movement to generate an optimum bone forming strain environment. There remains some concern regarding the level of control of length and rotation afforded by elastic nails. Pre-bending of elastic nails and the use of multiple nails are known to reduce the effect of angular and rotational forces on the fracture. In a study on simulated femoral fractures, Lee et al. [33] demonstrated that Ender nails maintained both length and rotational control of up to 40 % of body weight (with the assumption that body weight was 45 kg). This was true even in comminuted fracture patterns. This suggested that control of length and rotation in these fractures was sufficient and that patients could be allowed to mobilise early with the use of these devices [7].

Elastic nails offer a management option that is minimally invasive and allows natural bone healing by callus formation with a negligible re-fracture rate [7]. Retrieval of metalwork is simple and is carried out usually at 6 months facilitating an early return to social function and education. Elastic nailing has some disadvantages. Poor outcomes have been reported in larger children as well as those with comminuted fractures [34]. Narayanan et al. [35] and Sink et al. [36] reported an increased risk of shortening and malunion in length unstable fractures. For fractures that are axially unstable, endcaps may be used. These act by gripping the cortex and controlling shortening of the fracture [15] and prevent protrusion, a described complication in the literature [37].

#### External fixation

External fixation is a straightforward, technically easy method of stabilising femoral fractures. External fixators were first used in the management of paediatric femoral fractures in the late 1970s and became popular in the late 1980s to mid-1990s. A number of publications have reported excellent results with minimal complications [25, 38–40].

Despite allowing an early return of function, external fixators can lead to longer union times than elastic nailing or plating. Union of femoral fractures with external fixation takes a minimum of 8 weeks [41, 42], and some authors recommend leaving the external fixator on for up to 12 weeks [43]. Late dynamisation was thought to allow quicker healing but was found untrue, and less rigid frames should be used from the beginning of treatment. The use of external fixators carries the risk of delayed union, pin site infection, malalignment and refracture [43, 44]. The incidence of refracture varies greatly in the literature.

### The older child and the adolescent

In this group, operative management is favoured. The use of traction or casting is impractical as these methods cannot control the fracture fragments adequately and time to union is longer than in the younger groups. Intramedullary fixation is the mainstay of treatment with the decision whether or not to use elastic nails or a locked intramedullary nail.

The key determinant is the size of the child. Some authors advocate a limit of 50–60 kg as a cut-off point [7], suggesting that larger children benefit from locked nailing. Others [34] suggest that the cut-off point should be lower. In a consecutive series of 234 fractures, it was found that radiographic malunion was five times more likely in children over 49 kg. It is important to note that in this series the fracture type was not considered a variable. In a study on heavier children (47–85 kg) [45], using weight-matched cohorts and observing length stable fractures, no statistically significant malunion or leg length discrepancy was observed when elastic nailing was compared with rigid nailing.

**Fig. 12:**
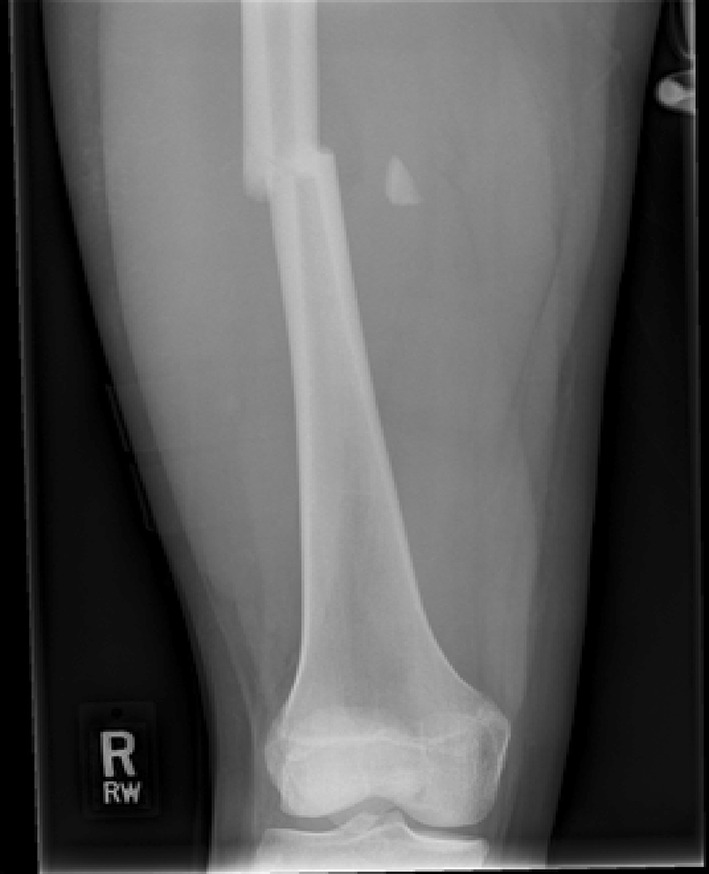
Treatment of a femoral diaphyseal fracture in a 16-year-old girl. Note is made of the subtle nuances such as the narrow canal and non-fused physis which must be considered in the management of these fractures

**Fig. 13:**
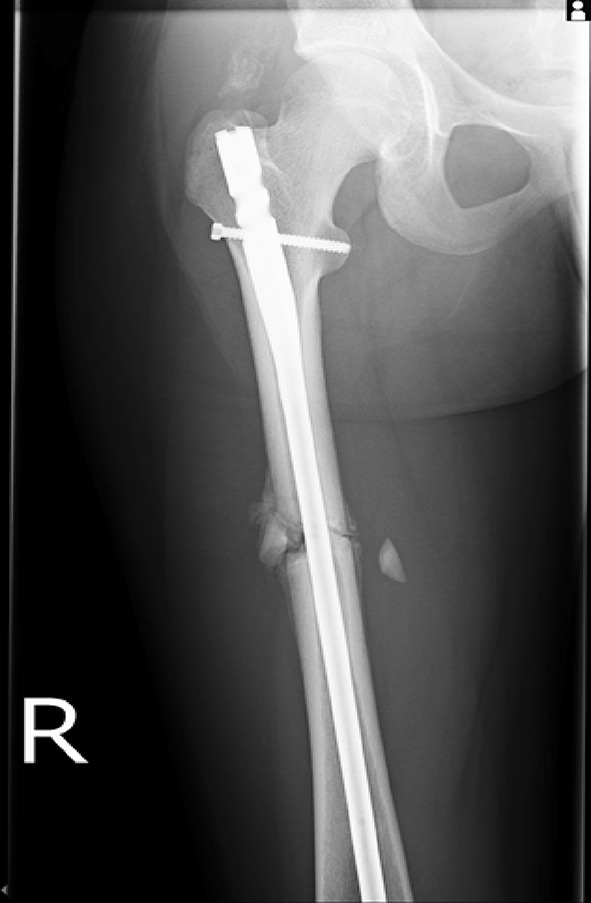
Treatment of a femoral diaphyseal fracture in a 16-year-old girl. Note is made of the subtle nuances such as the narrow canal and non-fused physis which must be considered in the management of these fractures

The use of adult type intramedullary nails in older children remains controversial. There is little doubt as to the efficacy of these devices in treating femoral fractures in adolescents [46–48] with length, alignment and union all easily achieved (Figs. 12, 13). The main risk associated with their use is the possibility of developing avascular necrosis of the femoral head; prior to physeal closure in the capital epiphysis, the blood supply to the femoral head originates from the region of the piriformis fossa which is coincidentally the entry portal of the standard intramedullary nail. Although there is no device on the market that can guarantee avoidance of this complication, some nails have been devised with alternative trochanteric entry points [49, 50]. Unfortunately, these have been associated with proximal growth disturbance in the femur related to damage to the trochanteric apophysis [51, 52].

The true incidence of AVN with adult nails remains unknown and is largely dependent on the technique used. A recent review [53] published in 2011 looked at data from 19 retrospective studies. Each technique was noted to have different rates of AVN. The piriformis entry group (comprising of 239 patients) had a 2 % AVN rate, the trochanteric entry point group (139 patients) had a rate of 1.4 %, and the lateral entry point group (80 Patients) had none suggesting that the lateral entry point is safer. It is important to note that the lateral entry point group was also the smallest.

Intramedullary nails designed specifically to cater for the anatomy of adolescents have been developed from the design of the original Kuntscher nail. Factoring in a better understanding of paediatric femoral anatomy, bony architecture as well as implant materials and metallurgy, a new design of a fatigue-resistant multiplanar rigid nail has emerged, which shows promising preliminary results [54].

Although the use of external fixators and submuscular plating remains an option in this age group, there is little research into the benefits of their use specifically in adolescents. In a comparative cohort study of different types of fixation, external fixation had the worst record for loss of reduction and malunion, even after adjusting for prognostic patient and fracture characteristics [55]. Nevertheless, a role remains for these methods of treatment, particularly in the multiple trauma setting (when external fixators retain their usefulness). There is an association between malunion and the use of nails in fractures with a significant degree of comminution (>25 %) [35].

### Open Fractures

Open femoral fractures are usually associated with high-energy trauma [56]. There are established protocols for management which include early collaboration between orthopaedics and plastic surgery [57]. The extent of the soft tissue injury will dictate the choice of implant used. External fixators remain a standard in the management of most open fractures. They allow for a minimally invasive technique with pins placed well away from the zone of injury. If an open wound can be closed primarily, then internal fixation may be appropriate. Elastic femoral nails rely on the integrity of the soft tissues around the injury to function correctly; therefore, severe open fractures with extensive soft tissue loss will be less stable when managed with elastic nailing than with other methods [7].

### Paediatric femoral fractures in the polytrauma setting

The optimal femoral fracture management in polytrauma depends on the age of the child and the severity of other injuries. Recent trends are early surgical stabilisation. There is evidence to suggest that early stabilisation in these patients leads to a lower complication rate from shorter periods of ventilatory support and intensive care unit stay [58]. Early stabilisation has also been shown to lead to a shorter hospital stay and fewer complications related to immobilisation [59]. Although there is some evidence to contradict these findings [60], a consensus remains that femoral trauma should be fixed surgically as soon as the child’s condition allows.

Casts, hip spicas and traction may be used as temporary measures until patients are fit enough for surgery but should be avoided in the treatment of open wounds and pressure areas. Patients with head injuries may be unsuitable for traction or casting as they will not tolerate such measures due to problems from cerebral irritation and muscle spasticity.

Elastic nailing may be advantageous as both antegrade and retrograde nails can be used to avoid operating in the zones of injury. The nursing care of patients with elastic nails is simpler. Contraindications to its use will include open or severely comminuted fractures. External fixators can offer a simple alternative means of treating femoral fractures nursed in the intensive care setting, but complications associated with external fixator use are more common in the multiply injured child [58].

## Discussion

The treatment of diaphyseal femoral fractures in children remains controversial as there are a number of effective treatment modalities. The lack of strong evidence to support one treatment form over another is reflected in the guidelines released by the American Academy of Orthopaedic Surgeons published in 2010 [61]. Most of the recommendations (10/14) within this guideline are based on level 4 or 5 evidence.

Trends in treatment have also varied historically. Presently, intramedullary elastic nailing is considered the treatment of choice in children aged 5–11; level III evidence exists to support this. Treatment for those outside the middle spectrum of age and weight veers towards non-operative management in the young and the use of locked intramedullary nailing in the older, heavier cohort. The American Academy report notes that rigid trochanteric entry nailing, submuscular plating and flexible intramedullary nailing are treatment options for children aged eleven to skeletal maturity (level III evidence). Early spica casting or traction with delayed spica casting for children aged 6 months to 5 years (with <2 cm of shortening) is the only form of treatment which is supported by level II evidence [61].

Irrespective of trends (historical or otherwise) in treatment, a decision on the type of fixation used should be based on the current evidence available. Where controversy exists, other factors must be considered. In the polytrauma setting, other factors such as open wounds and physiology may influence the modality of treatment and must be considered a separate pathway in any treatment algorithm.

The familiarity of the surgeon with a treatment modality and the required equipment is relevant; studies on technically demanding operations (Elastic Nailing) report that up to 75 % of all complications occur due to surgical inexperience [11].

**Fig. 14:**
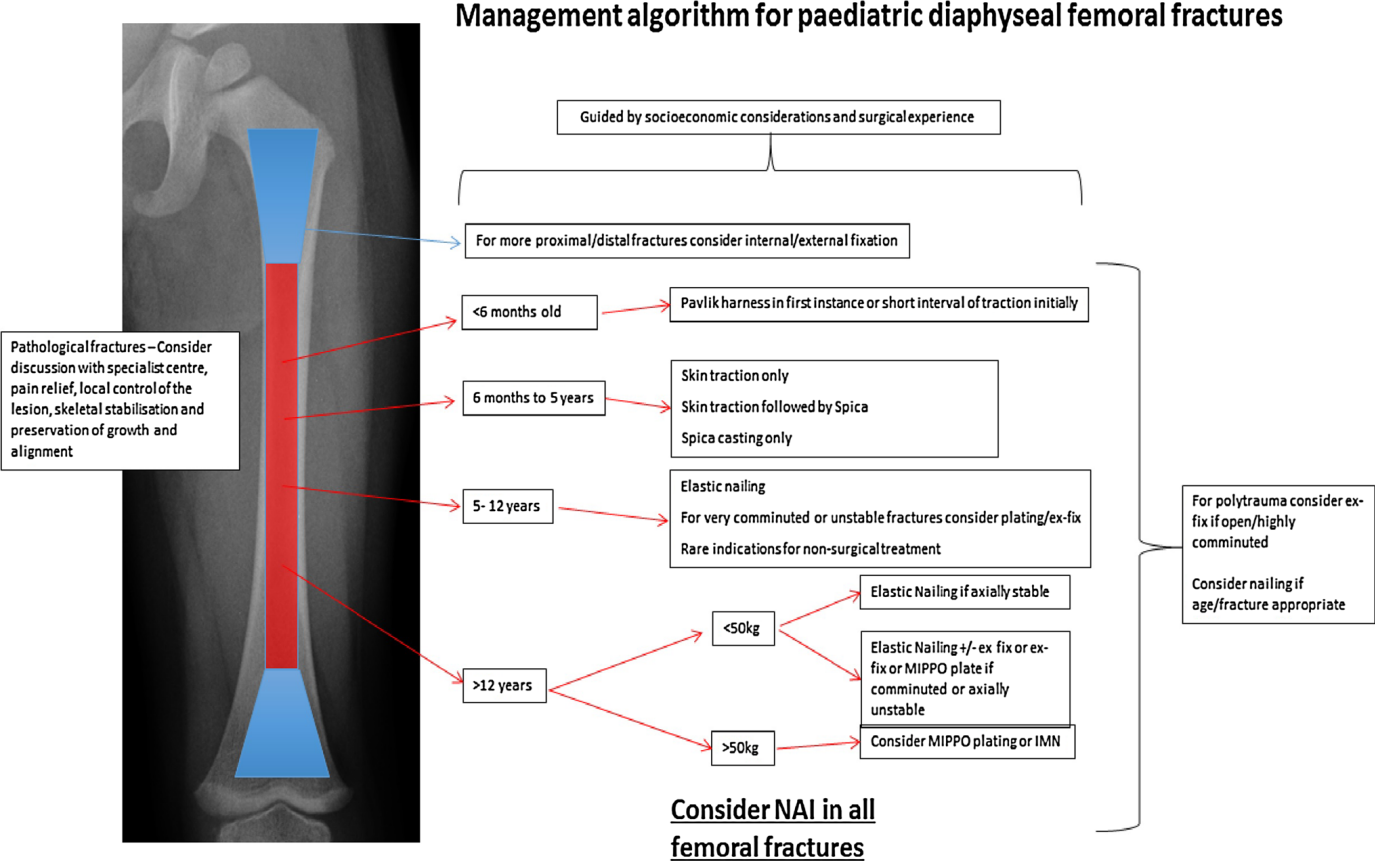
Algorithm summarising the management pathways for paediatric femoral diaphyseal fractures

Unfortunately, cost is a consideration particularly in a public healthcare setting. The cost difference, arising from surgery, implants and that of nursing care and length of admission, of two equally effective treatment modalities, may influence a patient’s management. The existing evidence and the complex socioeconomic considerations that apply in the modern era lead us to propose the following treatment algorithm (Fig. 14). It provides a clear pathway indicating the preferred modalities of treatment in each instance based on the current evidence available. Within the algorithm are decisions influenced by our experience and the facilities available in a level 1 major trauma centre.
